# Supplementary material for: Wafer-scale high-κ dielectrics for two-dimensional circuits via van der Waals integration
Source: Nat Commun. 2023 Apr 24;14:2340. doi: 10.1038/s41467-023-37887-x (PMC10125989; doi:10.1038/s41467-023-37887-x)
Supplement: Supplementary file 1 — Supplementary Information [file 41467_2023_37887_MOESM1_ESM.pdf]

Supplementary Information for

Wafer-scale high-κ dielectrics for two-dimensional circuits via van der Waals integration

Zheyi Lu[1], Yang Chen[1], Weiqi Dang[2], Lingan Kong[1], Quanyang Tao[1], Likuan Ma[1], Donglin Lu[1], Liting Liu[1], Wanying Li[1], Zhiwei Li[1], Xiao Liu[1], Yiliu Wang[1], Xidong Duan[2], Lei Liao[1], Yuan Liu[1,*]

[1]Key Laboratory for Micro-Nano Optoelectronic Devices of Ministry of Education, School of Physics and Electronics, Hunan University, Changsha 410082, China.

[2]Hunan Key Laboratory of Two-Dimensional Materials, State Key Laboratory for Chemo/Biosensing and Chemometrics, College of Chemistry and Chemical Engineering, Hunan University, Changsha 410082, China.

[*]Corresponding author. E–mail: yuanliuhnu@hnu.edu.cn

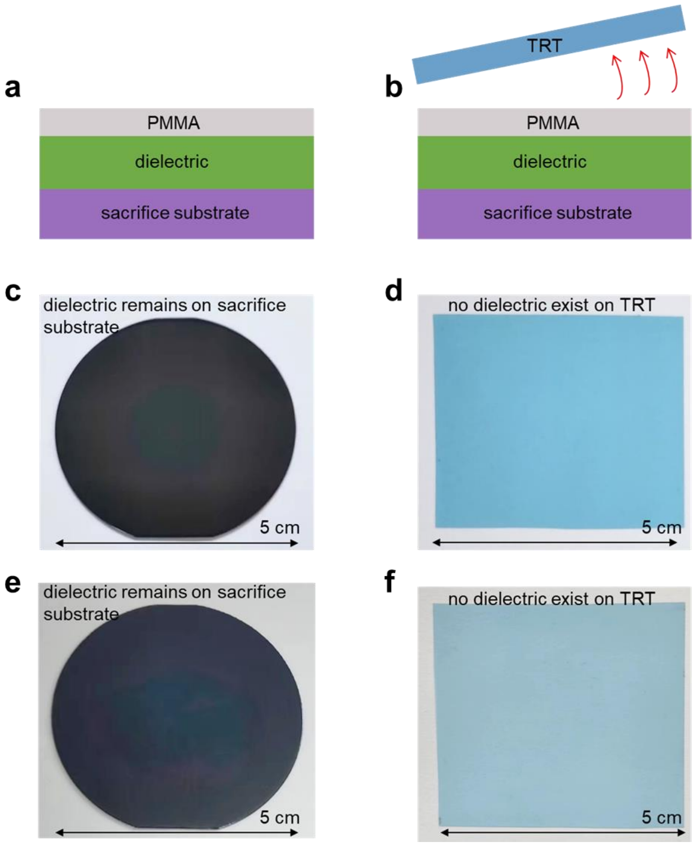

Supplementary Fig. 1. Dielectric peeling test using other polymers as the substrate. a, b, Schematic illustration of dielectric deposited on PMMA buffer layer the its peeling test. **c, -f** Optical images of sacrifice substrate (PMMA buffer layer for **c, d** and PPC buffer layer for **e, f**) and thermal release tape after the peeling test, where dielectric still remains on the sacrifice substrate and can not be peeled off.

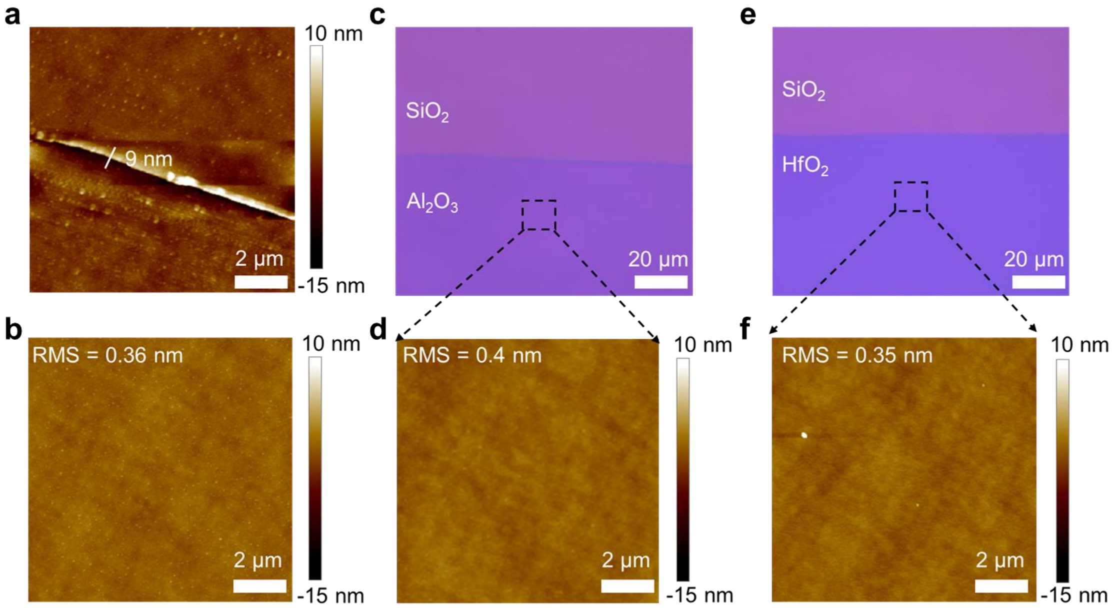

Supplementary Fig. 2. AFM characterization of PVA film and transferred dielectric. a, AFM measurement of the PVA film edge, demonstrating a thickness of 9 nm. **b,** AFM measurement of the PVA film surface, exhibiting a small RMS roughness of 0.36 nm. **c,** Optical image of transferred Al_2O_3. **d,** AFM measurement of the transferred Al_2O_3, with a small surface roughness of 0.4 nm. **e,** Optical image of transferred HfO_2. **f,** AFM measurement of the transferred HfO_2, with a small surface roughness of 0.35 nm.

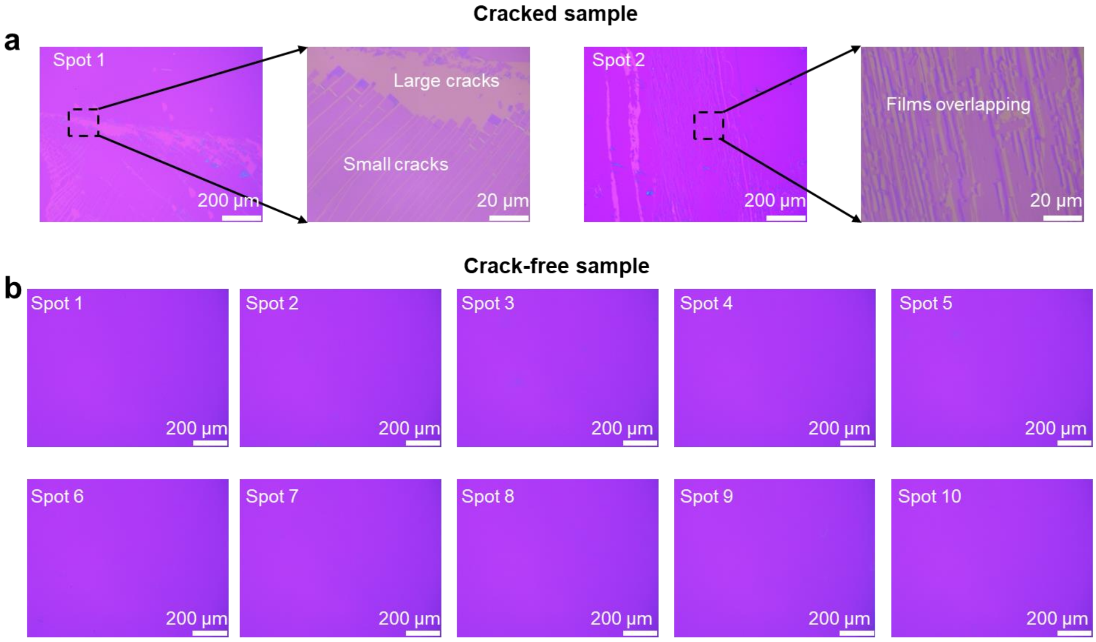

Supplementary Fig. 3. Optical images of 2-inch wafer Al_2O_3 without cracks and another control sample with cracks. **a**, Optical images of control sample by intentionally introducing large strain during lamination process, where the cracks can be clearly observed. **b**, Optical images of 10 different locations of crack-free Al_2O_3 film, demonstrating flat surface without clear cracks.

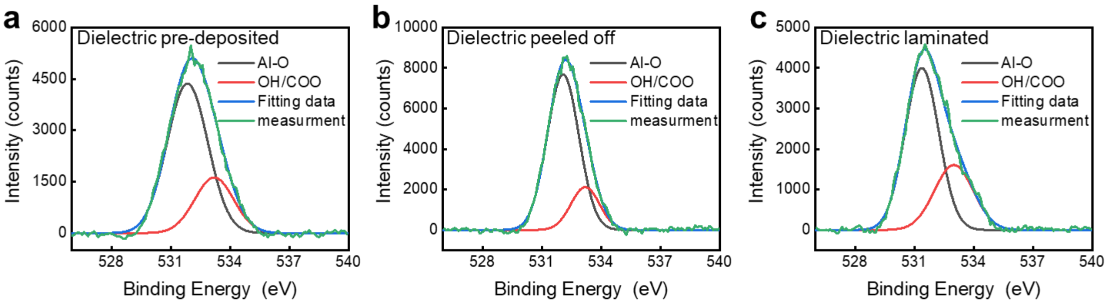

Supplementary Fig. 4. The XPS spectra of Al_2O_3 (O1s) at every transfer step. a-c, The XPS spectra of Al_2O_3 at different fabrication steps, including dielectric pre-deposited step (**a**), dielectric peeled off step (**b**), as well as dielectric laminated step(**c**).

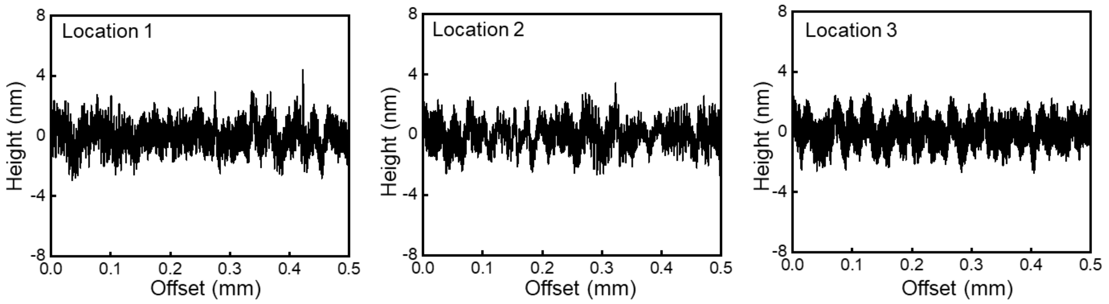

Supplementary Fig. 5. One-dimensional height mapping of the Al_2O_3 film using stair-meter for three different locations, demonstrating flat surface across larger area.

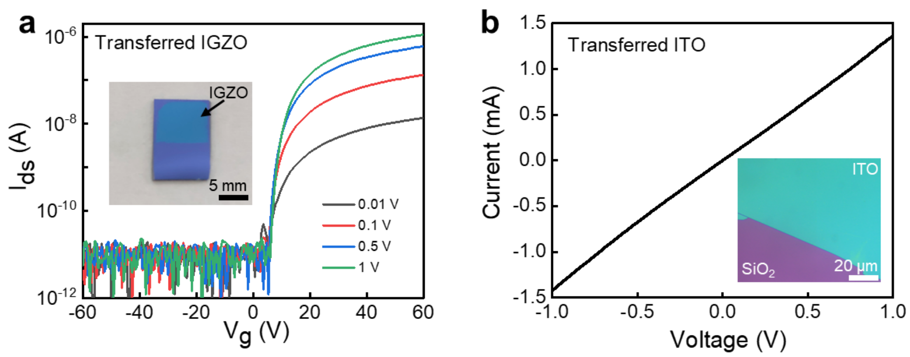

Supplementary Fig. 6. Transfer of other oxide material using vdW process. a, The optical images (inset) of transferred IGZO and the corresponding transistor behaviors using back gate. **b,** The optical images (inset) of transferred ITO and the corresponding conducting behaviors using two terminal measurement.

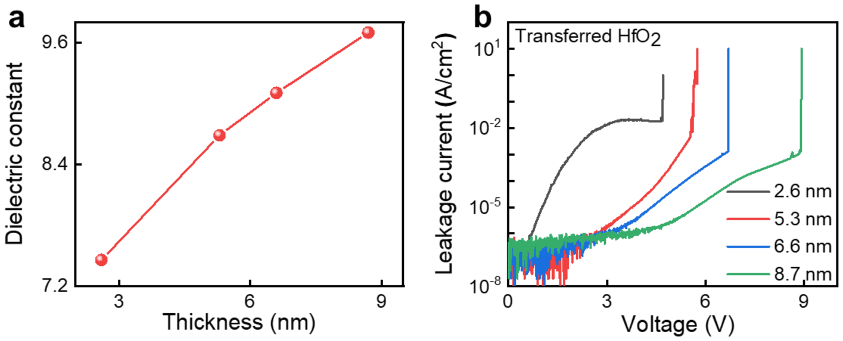

Supplementary Fig. 7. Dielectric properties of transferred HfO_2 films. a, Dielectric constant of transferred HfO_2 films with various thickness. **b,** The leakage current density of transferred HfO_2.

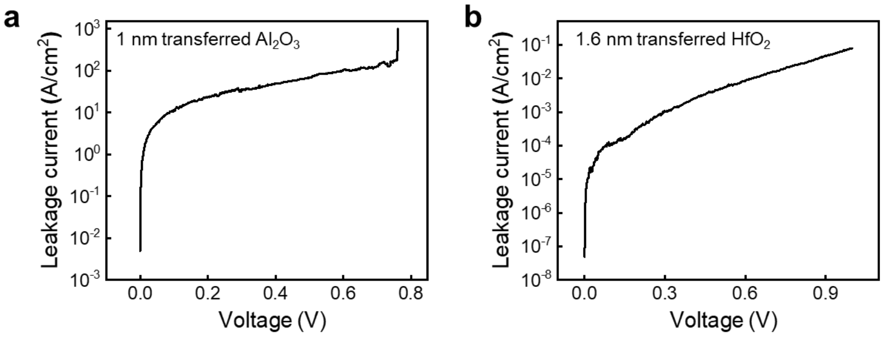

Supplementary Fig. 8. Leakage current of ultrathin transferred oxide films. a, b, Leakage current density of thinner transferred Al_2O_3 (**a**, 1 nm thick) and HfO_2 (**b**, 1.6 nm thick) films.

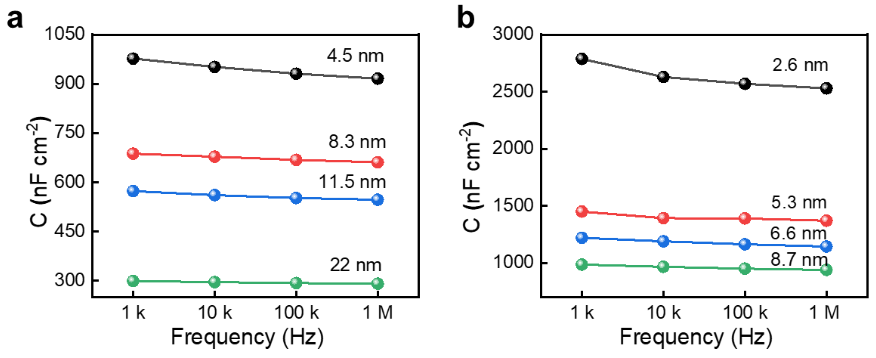

Supplementary Fig. 9. Capacitance of transferred oxide films with various frequency. a, The measured capacitance as a function of measurement frequency for transferred Al_2O_3 with various thickness. **b,** The measured capacitance as a function of measurement frequency for transferred HfO_2 with various thickness.

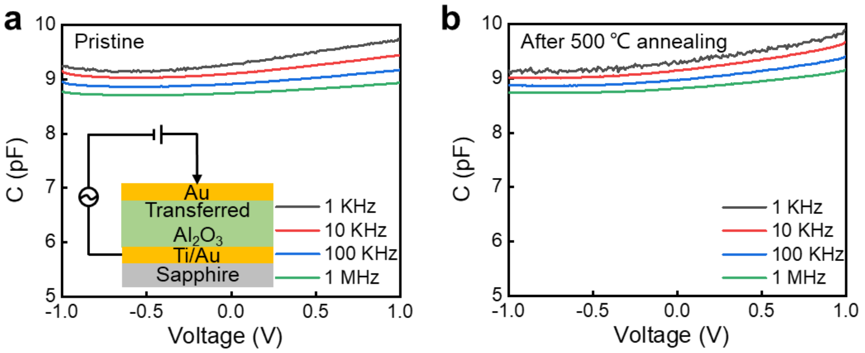

Supplementary Fig. 10. Thermal stability of vdW dielectric. a, *C-V* measurement of as-fabricated vdW Al_2O_3 films. **b**, *C-V* measurement vdW Al_2O_3 films after 500 °C annealing.

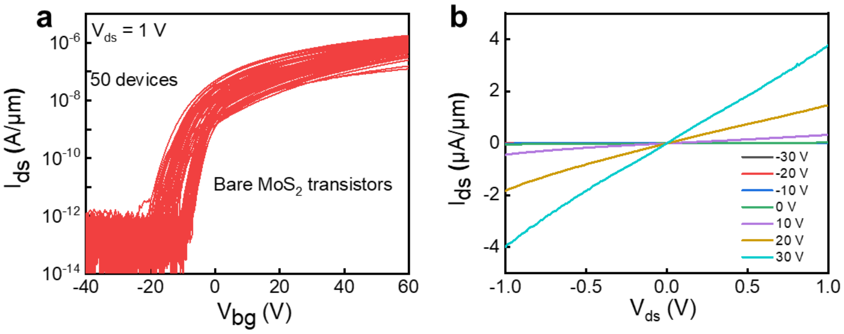

Supplementary Fig. 11. Electrical characterization of CVD-MoS_2 transistors array. a, I_{ds}-V_g transfer curves of back gate CVD-MoS_2 transistors array measured at 1 V bias voltage. **b,** The I_{ds}-V_{ds} output curves of a typical MoS_2 transistor.

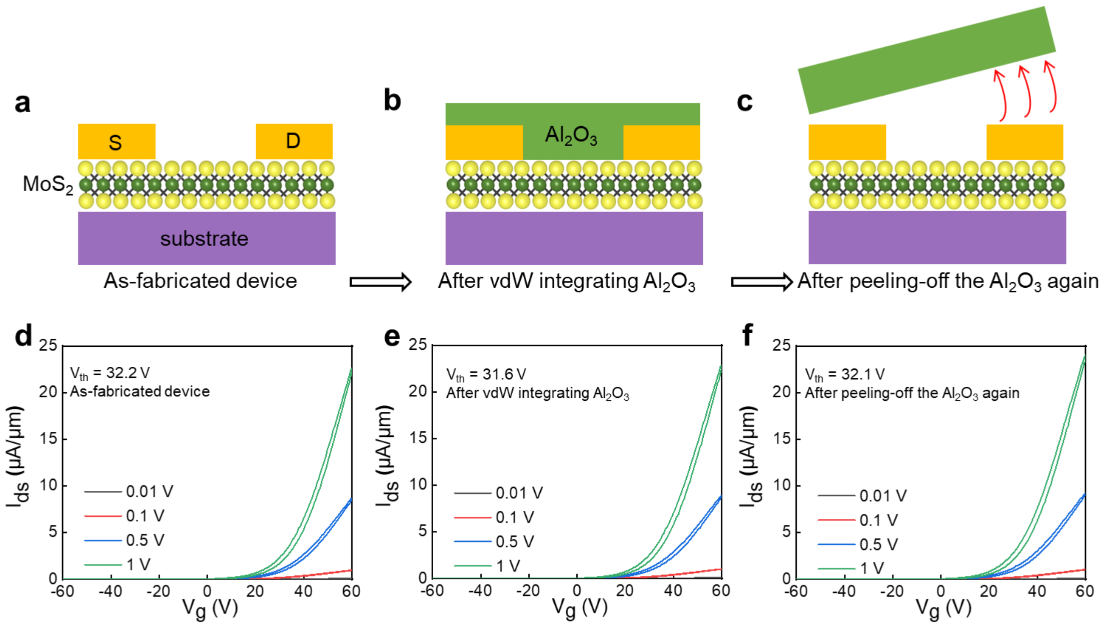

Supplementary Fig. 12. Electrical characterization of a MoS_2 FET integrated and removed Al_2O_3 dielectric. a-c, Schematics of MoS_2 FET with Al_2O_3 dielectric vdW integrating and peeling-off. **d** I_{ds}-V_g transfer curves of an as-fabricated MoS_2 transistor, demonstrating a threshold voltage of 32.2 V. **e**, The I_{ds}-V_g transfer curves after laminated 10 nm thick Al_2O_3 dielectric on top of MoS_2 FET. The threshold voltage shifts a small value of 0.6 V, and could be largely attributed to the change of dielectric environment. **f**, The I_{ds}-V_g transfer curves of the same device after peeling-off the integrated dielectric, where the threshold voltage restores to their originally state, suggesting the intrinsic MoS_2 channel is not impacted during the dielectric integration process.

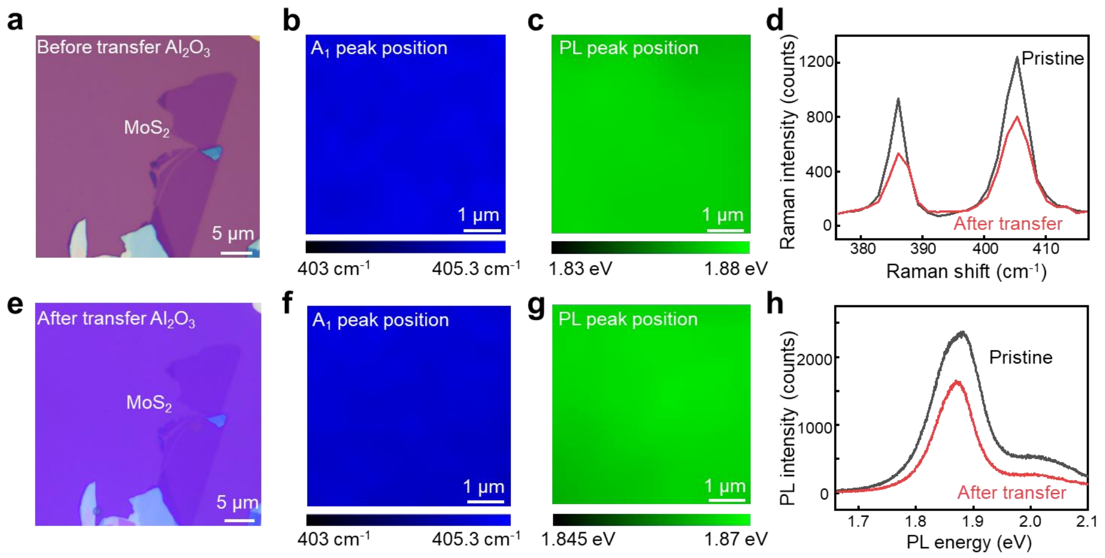

Supplementary Fig. 13. The Raman and PL spectra of monolayer MoS_2 before and after dielectric integration. a, Optical image of monolayer MoS_2 as-exfoliated on SiO_2 substrate. **b**, **c**, Raman mapping (A_1' peak position) and PL mapping of the monolayer MoS_2. **d**, The Raman spectra of monolayer MoS_2 before and after transferred Al_2O_3 dielectric. **e**, Optical image of monolayer MoS_2 after integrating Al_2O_3 dielectric. **f**, **g**, Raman mapping (A_1' peak position) and PL mapping of the monolayer MoS_2. **h**, The Raman spectra of monolayer MoS_2 before and after transferred Al_2O_3 dielectric.

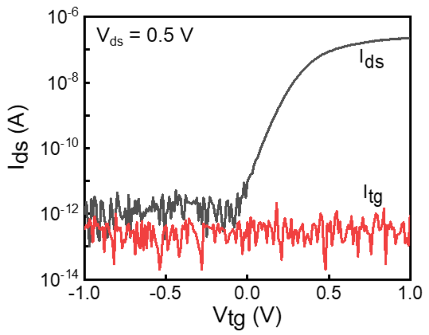

Supplementary Fig. 14. I_{ds}-V_{tg} transfer curves and gate leakage current of top-gate MoS_2 transistors fabricated by laminating 4.5 nm Al_2O_3, exhibiting high on/off ratio and low gate leakage current.

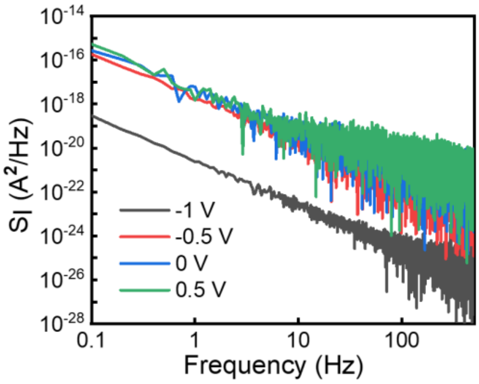

Supplementary Fig. 15. Noise spectra as a function of frequency with different gate bias at V_{ds} = 1 V.

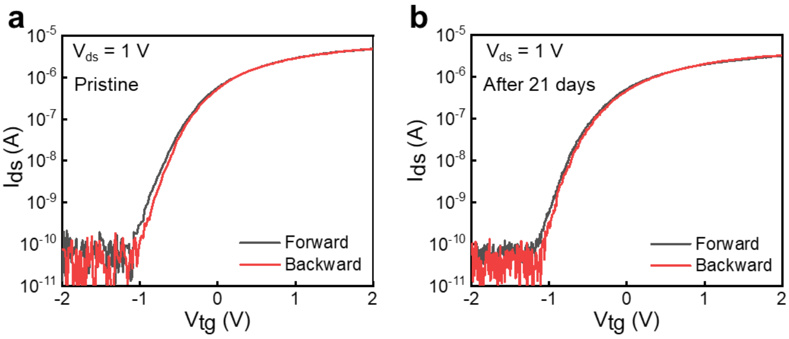

Supplementary Fig. 16. Long-term stability of top-gate MoS_2 device. a, I_{ds}-V_{tg} transfer curves of as-fabricated MoS_2 transistor. **b**, I_{ds}-V_{tg} transfer curves of MoS_2 transistor measured after 3 weeks storage, demonstrating negligible change.

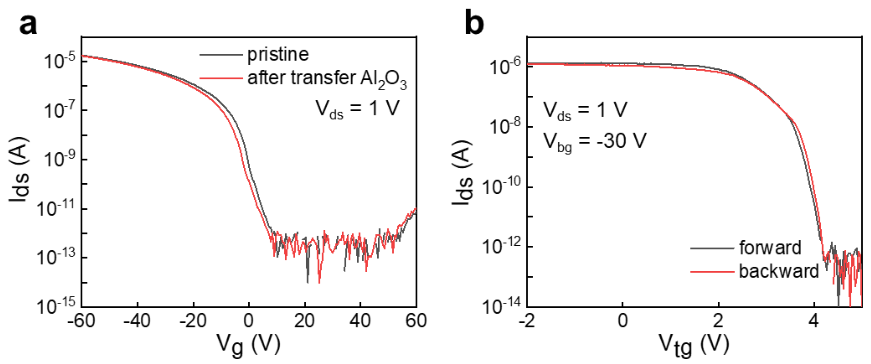

Supplementary Fig. 17. The universality of our vdWs dielectric integration method. a, I_{ds}-V_g transfer curves of WSe_2 transistor before and after integrating Al_2O_3 insulator films. **b,** I_{ds}-V_{tg} transfer curves of top-gate WSe_2 transistor fabricated by transferred 10 nm Al_2O_3.

Supplementary Table 1. Comparison of our vdW process with other dielectric integration method.

Dielectric	Method	scalability	Hysteresis (mV)	EOT (nm)	SS (mV/dec)	Ref.
CaF_2	CVD	Micrometer	35	0.9	93	1
$SrTiO_3$	transfer	Centimeter	42	1	71	2
β-Bi_2SeO_5	Plasma oxidation	Micrometer	60	0.5	62	3
PTCDA/HfO_2	CVD/ALD	Large-area	10	2	64	4
Al_2O_3	ALD	Wafer-scale	452	23	138	5
HfO_2	ALD	Wafer-scale	125	10.5	233	6
Sb_2O_3	Thermal evaporation	Wafer-scale	10	3.3	68	7
ZrO_2	ALD	Wafer-scale	155	2.3	90	8
Al_2O_3 or HfO_2	transfer	Wafer-scale	10	1.3	68	This Work

Supplementary References

1. Illarionov, Y. Y. et al. Ultrathin calcium fluoride insulators for two-dimensional field-effect transistors. *Nat. Electron.* **2**, 230-235 (2019).

2. Huang, J.-K. et al. High-κ perovskite membranes as insulators for two-dimensional transistors. *Nature* **605**, 262–267 (2022).

3. Zhang, Y. et al. A single-crystalline native dielectric for two-dimensional semiconductors with an equivalent oxide thickness below 0.5 nm. *Nat. Electron.* **5**, 643-649 (2022).

4. Li, W. et al. Uniform and ultrathin high-κ gate dielectrics for two-dimensional electronic devices. *Nat. Electron.* **2**, 563–571 (2019).

5. Li, T. et al. Electrical performance of multilayer MoS_2 transistors on high-κ Al_2O_3 coated Si substrates. *AIP Adv.* **5**, 057102 (2015).

6. Wen, M. et al. Effects of annealing on electrical performance of multilayer MoS_2 transistors with atomic layer deposited HfO_2 gate dielectric. *Appl. Phys. Express* **9**, 095202 (2016).

7. Liu, K. et al. A wafer-scale van der Waals dielectric made from an inorganic molecular crystal film. *Nat. Electron.* **4**, 906-913 (2021).

8. Chang, W. H. et al. ALD-ZrO_2 gate dielectric with suppressed interfacial oxidation for high performance MoS_2 top gate MOSFETs. *Jpn. J. Appl. Phys.* **60**, SBBH03 (2021).
