# Supplementary material for: Wafer-scale high-κ dielectrics for two-dimensional circuits via van der Waals integration
Source: Nat Commun. 2023 Apr 24;14:2340. doi: 10.1038/s41467-023-37887-x (PMC10125989; doi:10.1038/s41467-023-37887-x)
Supplement: Supplementary file 3 — Description of Additional Supplementary Files [file 41467_2023_37887_MOESM3_ESM.docx]

**Description of Additional Supplementary Files**

File Name: Supplementary Movie 1

Description: Movie of dielectric transfer and lamination process: including dielectric peeling-off process, buffer layer etching process, dielectric lamination process, and the final sample demonstration.
